# Interpretation of vaginal metagenomic characteristics in different types of vaginitis

**DOI:** 10.1128/msystems.01377-23

**Published:** 2024-02-16

**Authors:** Jiarong Song, Xue Dong, Yue Lan, Yunwei Lu, Xu Liu, Xuena Kang, Zhonglu Huang, Bisong Yue, Yu Liu, Wenjin Ma, Libo Zhang, Haijun Yan, Miao He, Zhenxin Fan, Tao Guo

**Affiliations:** 1 Key Laboratory of Bioresources and Ecoenvironment (Ministry of Education), College of Life Sciences, Sichuan University, Chengdu, China; 2 Department of Gynecology and Obstetrics, West China Second Hospital, Sichuan University, Chengdu, China; 3 Meishan Women and Children’s Hospital, Meishan, Sichuan, China; 4 Institute of Blood Transfusion, Chinese Academy of Medical Sciences, Chengdu, Sichuan, China; 5 Chenghua District Maternal and Child Health Hospital, Chengdu, Sichuan, China; 6 Renshou County People’s Hospital, Renshou, Sichuan, China; 7 Meishan Traditional Chinese Medicine Hospital, Meishan, Sichuan, China; Cleveland Clinic, Cleveland, Ohio, USA

**Keywords:** bacterial vaginosis, vulvovaginal candidiasis, metagenomics, vaginal flora, *Lactobacillus*

## Abstract

Although vaginitis is closely related to vaginal microecology in females, the precise composition and functional potential of different types of vaginitis remain unclear. Here, metagenomic sequencing was applied to analyze the vaginal flora in patients with various forms of vaginitis, including cases with a clue cell proportion ranging from 1% to 20% (Clue1_20), bacterial vaginitis (BV), vulvovaginal candidiasis (VVC), and BV combined with VVC (VVC_BV). Our results identified *Prevotella* as an important biomarker between BV and Clue1_20. Moreover, a gradual decrease was observed in the relative abundance of shikimic acid metabolism associated with bacteria producing indole as well as a decline in the abundance of *Gardnerella vaginalis* in patients with BV, Clue1_20, and healthy women. Interestingly, the vaginal flora of patients in the VVC_BV group exhibited structural similarities to that of the VVC group, and its potentially functional characteristics resembled those of the BV and VVC groups. Finally, *Lactobacillus crispatus* was found in high abundance in healthy samples, greatly contributing to the stability of the vaginal environment. For the further study of *L. crispatus*, we isolated five strains of *L. crispatus* from healthy samples and evaluated their capacity to inhibit *G. vaginalis* biofilms and produce lactic acid *in vitro* to select the potential probiotic candidate for improving vaginitis in future clinical studies. Overall, we successfully identified bacterial biomarkers of different vaginitis and characterized the dynamic shifts in vaginal flora between patients with BV and healthy females. This research advances our understanding and holds great promise in enhancing clinical approaches for the treatment of vaginitis.

**IMPORTANCE:**

Vaginitis is one of the most common gynecological diseases, mostly caused by infections of pathogens such as *Candida albicans* and *Gardnerella vaginalis*. In recent years, it has been found that the stability of the vaginal flora plays an important role in vaginitis. Furthermore, the abundant *Lactobacillus*-producing rich lactic acid in the vagina provides a healthy acidic environment such as *Lactobacillus crispatus*. The metabolites of *Lactobacillus* can inhibit the colonization of pathogens. Here, we collected the vaginal samples of patients with bacterial vaginitis (BV), vulvovaginal candidiasis (VVC), and BV combined with VVC to discover the differences and relationships among the different kinds of vaginitis by metagenomic sequencing. Furthermore, because of the importance of *L. crispatus* in promoting vaginal health, we isolated multiple strains from vaginal samples of healthy females and chose the most promising strain with potential probiotic benefits to provide clinical implications for treatment strategies.

## INTRODUCTION

Vaginitis, a prevalent gynecological condition, represents a recurrent challenge for women worldwide. Clinically, it is characterized into bacterial vaginosis (BV), vulvovaginal candidiasis (VVC), and trichomonas vaginitis (TV) based on the nature of the infection (1, 2). Due to various factors, some women may experience multiple types of vaginitis simultaneously. Moreover, disruptions in vaginal flora due to BV have been associated with an elevated risk of human immunodeficiency virus (HIV) (3, 4) and human papilloma virus (HPV) infections (5). Clinical symptoms of BV commonly include an abundance of clue cells (vaginal epithelial cells heavily coated with bacteria) that make up more than 20% of the vaginal flora (6, 7). Interestingly, there are women who exhibit symptoms and have between 1% and 20% clue cells in their vagina yet do not meet the clinical criteria for a BV diagnosis (8, 9). The presence of clue cells in these instances provokes concerns regarding the health status of the vaginal flora.

Previously, the primary treatment options for BV and VVC involved the use of metronidazole and clindamycin (10–12). More recently, however, probiotics have gained increasing public acceptance due to their non-destructive impact on vaginal flora stability (13–15). *Lactobacillus* species, which represent the predominant bacteria in the vaginal flora of healthy females, produce metabolites to inhibit the colonization of pathogenic bacteria, such as *Gardnerella vaginalis*, and provide an acidic environment (pH 4.5) in the vagina (16, 17). Various studies have evaluated the effects of different *Lactobacillus* species in patients with vaginitis, including *Lactobacillus rhamnosus*, *Lactobacillus gasseri*, and *Lactobacillus crispatus* (18–20).

Many studies have explored the vaginal microbiota using advanced next-generation sequencing, with a particular focus on 16S rRNA sequencing analysis (2, 21–24). However, the accuracy of species-level annotation using 16S rRNA sequencing is limited (25). In contrast, metagenomic sequencing provides a more comprehensive and in-depth characterization of the complexity of the microbiome (26).

Here, we employed metagenomic sequencing to investigate the structural and variances of the functional potential in the vaginal microbiota between healthy females and those with different types of vaginitis. This allowed us to identify specific biomarkers associated with different forms of vaginitis. We further conducted a comparative analysis of samples contained 1%–20% clue cells and those from healthy women and BV patients to clarify their relationships and differences. We also investigated the similarities and differences between individuals with BV, VVC, and BV combined with VVC. By isolating and evaluating high-performing *Lactobacillus* strains from the vagina of healthy females, this study aims to provide the evidence for generating hypotheses for potential treatment strategies.

## RESULTS

### Significant structural differences in vaginal flora between healthy women and patients with vaginitis

**Fig 1:**
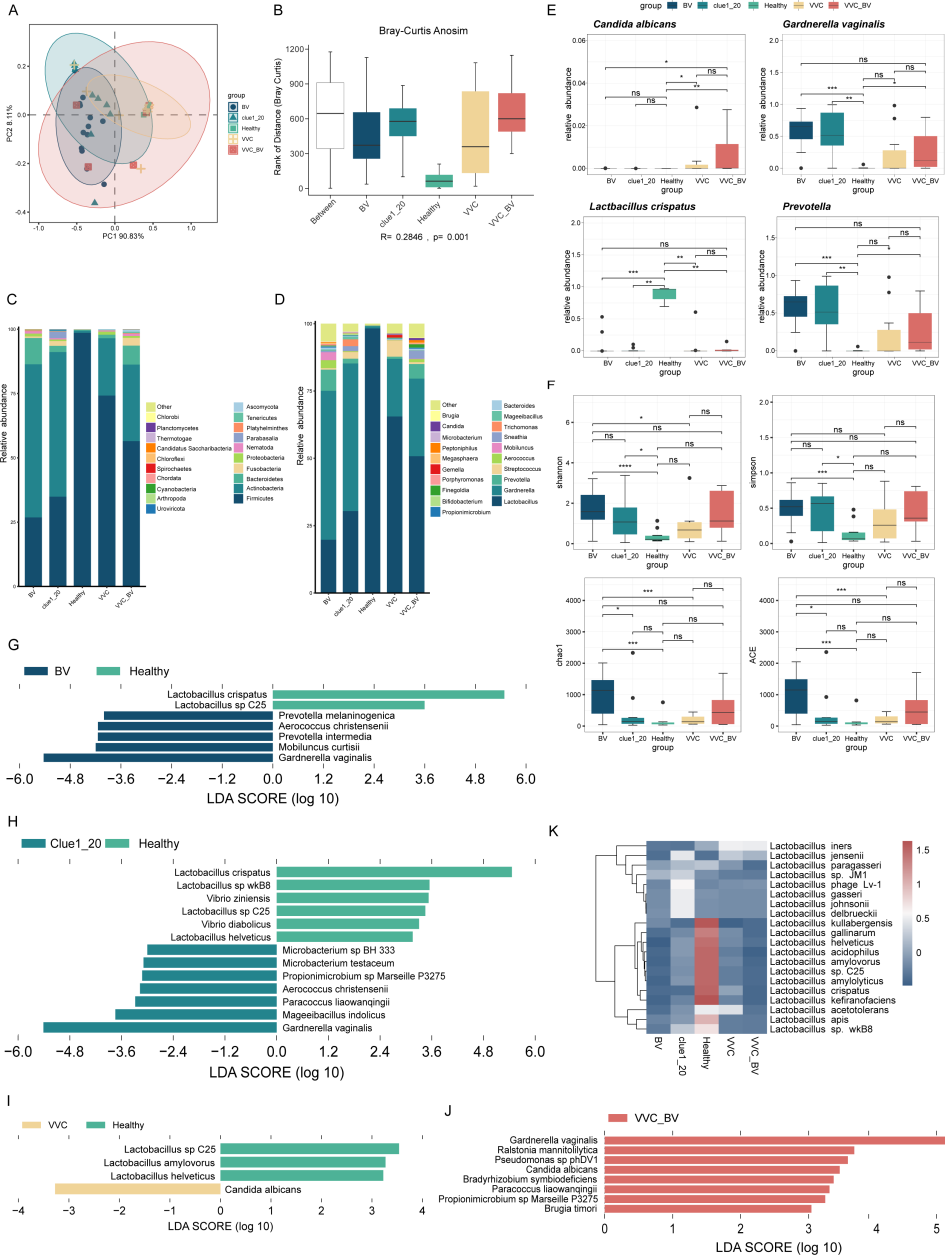
The comparisons of the vaginal microbiome composition between healthy females and patients with different types of vaginitis. (**A and B**) Phylum-level β-diversity among the five groups based on principal coordinate analysis (PCoA) and Bray-Curtis analysis of similarities (Bray-Curtis Anosim) (*R* = 0.2846*, P* = 0.001). (**C and D**) Top 20 most abundant bacteria in five groups at the phylum (**C**) and genus (**D**) levels. (**E**) The comparison of the relative abundance of *Candida albicans*, *Gardnerella vaginalis*, *Lactobacillus crispatus* and *Prevotella* among the five groups. (**F**) The differences of α-diversity in the five groups. (**G**) The different microbes between the healthy and BV group by LEfSe (*P* < 0.05 and LDA > 3.5). (**H**) The different microbes between the healthy and Clue1_20 group by LEfSe (*P* < 0.05 and LDA > 3). (**I**) The different microbes between the healthy and VVC group by LEfSe (*P* < 0.05 and LDA > 3). (**J**) The different microbes between the healthy and VVC_BV group by LEfSe (*P* < 0.05 and LDA > 3). (**K**) The heatmap of the relative abundance of *Lactobacillus* contained in the five groups. ^ns^*P*-value >0.05, ^*^P-value < 0.05; ^**^P-value < 0.01; ^***^P-value < 0.001; ^****^P-value < 0.0001 in panels E and F.

Metagenomic sequencing was carried out on samples obtained from 41 patients with vaginitis and 8 healthy females, resulting in 226.56 Gb of raw data and 221.92 Gb of filtered clean data. The average number of raw reads sequenced per sample was 36,082,562, and the average number of non-host reads sequenced per sample was 3,324,968 (Tables S1 and S4). Based on metagenomic sequencing, the vaginal microbiota of the five groups was classified into 39 phyla, 816 genera, and 1,523 species. Results showed significant differences in the diversity of the vaginal microbiota between the healthy group and the BV, VVC, Clue1_20, and VVC_BV groups (*R* = 0.2846, *P* = 0.001; Fig. 1A and B). Notably, marked differences were observed between the healthy and BV and Clue1_20 groups or between the BV and VVC groups based on the Shannon index (*P* < 0.0001, *P* < 0.05, and *P* < 0.05, respectively; Fig. 1F). There were significant differences between the BV and healthy groups or the Clue1_20 and healthy groups based on the Simpson index (*P* < 0.001 and *P* < 0.05, respectively; Fig. 1F). Marked differences were observed between the BV and healthy and Clue1_20 and VVC groups based on the Chao1 and ACE indices (*P* < 0.001, *P* < 0.05, and *P* < 0.001, respectively; Fig. 1F). These findings indicated distinct variations in the vaginal microbiota of healthy individuals and patients with vaginitis. Furthermore, the vaginal microbiota in vaginitis patients displayed greater complexity compared to that of healthy women.

To gain a comprehensive understanding of the whole composition of the vaginal microbiome in the five groups, a vaginal microbiome profile was generated. At the phylum level, Firmicutes was dominant in the healthy group (98.58%), while Actinobacteria (59.54%), Firmicutes (26.81%), and Bacteroidetes (10.23%) were dominant in the BV group. Moreover, Actinobacteria and Firmicutes were dominant in the Clue1_20 group (56.20% and 34.89%, respectively), VVC group (22.30% and 74.18%, respectively), and VVC_BV group (29.74% and 56.48%, respectively) (Fig. 1C).

At the genus level, *Lactobacillus* (98.20%) was substantially more abundant in the healthy group compared to the other groups (Fig. 1D). Moreover, in the BV group, *Gardnerella* (55.34%) was the most abundant, followed by *Lactobacillus* (19.73%) and *Prevotella* (7.84%). In the Clue1_20 group, *Gardnerella* (54.80%) and *Lactobacillus* (30.40%) were the most abundant, followed by *Prevotella* (1.78%). In the VVC group, the vaginal flora was dominated by *Lactobacillus* (65.52%), *Gardnerella* (21.41%), and *Streptococcus* (6.02%), while *Lactobacillus* (50.76%), *Gardnerella* (28.81%), and *Prevotella* (5.42%) were dominant in the VVC_BV group (Fig. 1D). Specifically, at the species level of *Lactobacillus*, *L. crispatus* and *Lactobacillus iners* were the dominant *Lactobacillus* in the healthy group (66.58% and 29.34%, respectively), BV group (3.56% and 16.03%, respectively), Clue1_20 group (4.15% and 15.46%, respectively), VVC group (16.08% and 46.41%, respectively), and VVC_BV group (3.34% and 45.24%, respectively). The relative abundance of *Lactobacillus jensenii* in the Clue1_20, VVC, and VVC_BV groups was 3.45%, 2.71%, and 2.03%, respectively, but extremely low in the BV and healthy group. In the Clue1_20 group, the relative abundance of *Lactobacillus johnsonii* and *L. gasseri* was 4.16% and 2.04%, respectively, but extremely low in others. In addition, *Lactobacillus helveticus* (0.39%), *Lactobacillus amylovorus* (0.46%), and *Lactobacillus acidophilus* (0.10%) were richer in the healthy group than other groups (Fig. 1K).

We next conducted linear discriminant analysis (LDA) effect size (LEfSe) between each patient group and the healthy group to identify bacterial biomarkers. Significant changes were observed in seven microbes at the species level between the healthy and BV groups (LDA >3.5) (Fig. 1G). *G. vaginalis*, *Mobiluncus curtisii*, *Prevotella intermedia*, *Aerococcus christensenii*, and *Prevotella melaninogenica* were significantly enriched in the BV group, while *L. crispatus* and *Lactobacillus* sp. C25 were significantly enriched in the healthy group. Significant changes were observed in 13 microbes at the species level between the healthy and Clue1_20 groups (LDA >3) (Fig. 1H). *G. vaginalis*, *Mageeibacillus indolicus*, *Paracoccus liaowanqingii*, *A. christensenii*, *Propionimicrobium* sp. Marseille P3275, *Microbacterium testaceum*, and *Microbacterium* sp. BH 333 were significantly enriched in the Clue1_20 group, while *L. crispatus*, *Lactobacillus* sp. wkB8, *Vibrio ziniensis*, *Lactobacillus* sp. C25, *L. helveticus*, and *Vibrio diabolicus* were significantly enriched in the healthy group. Significant changes were observed in four microbes at the species level between the healthy and VVC groups (LDA >3) (Fig. 1I). *Candida albicans* was significantly enriched in the VVC group, while *L. amylovorus*, *L.* sp. C25, and *L. helveticus* were significantly enriched in the healthy group. Significant changes were observed in eight microbes at the species level between the healthy and VVC_BV groups (LDA >3) (Fig. 1J). *G. vaginalis*, *Ralstonia mannitolilytica*, *Pseudomonas* sp. phDV1, *C. albicans*, *Bradyrhizobium symbiodeficiens*, *P. liaowanqingii*, *Propionimicrobium* sp. Marseille P3275, and *Brugia timori* were significantly enriched in the VVC_BV group.

Based on the above results, we generated box plots to visualize differences in five bacterial biomarkers among the five groups (Fig. 1E). Compared to the healthy group, the relative abundance of *C. albicans* was remarkably higher in the VVC_BV group (*P* < 0.01) as well as in the VVC group (*P* < 0.05). There was a significant difference between the BV and VVC_BV groups (*P* < 0.05). Compared to the healthy group, the relative abundances of *G. vaginalis* and *Prevotella* were significantly higher in the BV (*P* < 0.001), Clue1_20 (*P* < 0.01), and VVC_BV groups (*P* < 0.05). Furthermore, results also showed that the relative abundance of *L. crispatus* was significantly higher in the healthy group compared to the BV, Clue1_20, VVC, and VVC_BV groups (*P* < 0.001, *P* < 0.01, *P* < 0.01, and *P* < 0.01, respectively; Fig. 1E). These findings corroborated our earlier observations.

### Relationship between percentage of clue cells and vaginal flora structure

**Fig 2:**
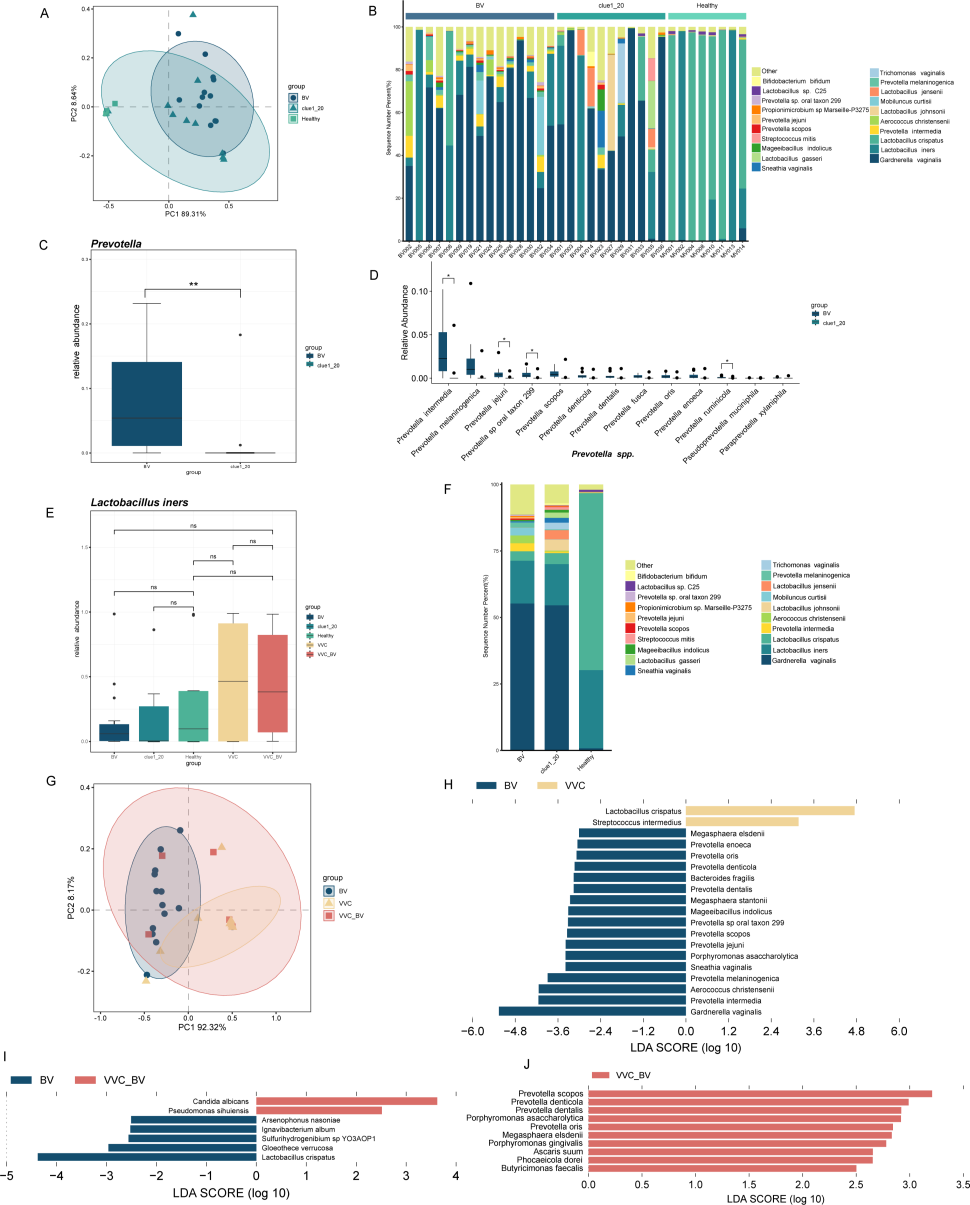
The relationships of the vaginal microbiome composition among the healthy, Clue1_20, and BV groups and the comparison of the vaginal microbiome composition among the BV, VVC, and VVC_BV groups. (**A**) The difference of β-diversity among the healthy, Clue1_20, and BV groups (Bray-Curtis Anosim, *R* = 0.3895*, P* = 0.001). (**B**) Top 20 most abundant bacteria at the species level in the healthy, Clue1_20, and BV groups. (**C**) The comparison of the relative abundance of *Prevotella* between the BV and Clue1_20 groups. (**D**) The box plot of the relative abundance of 13 species of *Prevotella* between the BV and Clue1_20 groups. (**E**) The comparison of the relative abundance of *Lactobacillus iners* among the five groups. (**F**) Top 20 most abundant bacteria at the species level in the healthy, Clue1_20, and BV groups (mean data). (**G**) The difference of β-diversity among the BV, VVC, and VVC_BV groups (Bray-Curtis Anosim, *R* = 0.3012*, P* = 0.001). (**H**) The different microbes between the BV and VVC groups by LEfSe (*P* < 0.05 and LDA >3). (**I**) The different microbes between the BV and VVC_BV groups by LEfSe (*P* < 0.05 and LDA >2.5). (**J**) The different microbes among the VVC and VVC_BV groups by LEfSe (*P* < 0.05 and LDA >3). ^ns^*P*-value >0.05, ^*^P-value <0.05; ^**^P-value <0.01; ^***^P-value <0.001 in panels C, D, and E.

Clue cells were vaginal epithelial cells that have *G. vaginalis* attached to their surfaces (7). Samples from women with 1%–20% clue cells in the vagina were collected and compared with samples from BV patients and healthy women. According to β-diversity analysis, the healthy group showed significant differences from the BV and Clue1_20 groups, while no significant differences were detected between the BV and Clue1_20 groups (*R* = 0.3895*, P* = 0.001; Fig. 2A).

At the family level, microbial community analysis revealed that differences in the prevalence of Prevotellaceae were the main distinction among the BV, Clue1_20, and healthy groups, with a notable increase in the BV group (7.78%) compared to the Clue1_20 (1.76%) and healthy groups (0.22%) (Fig. S1). Lactobacillaceae, also one of the main distinctions among the three groups, was markedly lower in the Clue1_20 (30.04%) and BV groups (19.84%) compared to the healthy group (98.25%) (Fig. S1). To further explore the differences between the BV and Clue1_20 groups, all Prevotellaceae bacteria detected in the samples were examined, and the overall relative abundance of them in the BV group was significantly higher than that in the Clue1_20 group (*P* < 0.01; Fig. 2C). Results demonstrated that the relative abundances of 4 of the 13 species were significantly higher in the BV group than in the Clue1_20 group, including *P. intermedia*, *Prevotella jejuni*, *Prevotella* sp. oral taxon 299, and *Prevotella ruminicola* (*P* < 0.05; Fig. 2D). Moreover, at the species level of *Lactobacillus*, the healthy group was dominated by *L. crispatus* (66.58%) and *L. iners* (29.34%), while the BV (16.04%) and Clue1_20 (15.46%) groups were dominated by *L. iners* (Fig. 2B and F). Furthermore, there was no significant difference of the relative abundance of *L. iners* in five groups (*P* > 0.05; Fig. 2E). On the contrary, *G. vaginalis* was dominant in the BV (55.23%) and Clue1_20 (54.55%) groups; however, its relative abundance was far lower in the healthy group (0.82%) (Fig. 2F). The above findings demonstrated that the relative abundance of *Lactobacillus* was highest in the healthy group, followed by the Clue1_20 and BV groups, while *G. vaginalis* showed the opposite trend.

### Structural comparison of vaginal flora in patients with different types of vaginitis

Results of principal coordinate analysis (PCoA) showed significant differences between the BV and VVC groups but no significant differences between the VVC and VVC_BV groups or between the BV and VVC_BV groups (*R* = 0.3012, *P* = 0.001; Fig. 2G). Based on LEfSe analysis, the BV and VVC groups of 19 microbes were significantly different at the species level (LDA >3; Fig. 2H). *L. crispatus* and *Streptococcus intermedius* were higher in the VVC group, while *G. vaginalis*, *P. intermedia*, *A. christensenii*, *P. melaninogenica*, *P. asaccharolytica*, and *Sneathia vaginalis* were higher in the BV group. The BV and VVC_BV groups of seven microbes were significantly different at the species level (LDA >2.5; Fig. 2I). *C. albicans* and *Pseudomonas sihuiensis* were enriched in the VVC_BV group, while *L. crispatus*, *Gloeothece verrucosa*, *Sulfurihydrogenibium* sp. YO3AOP1, *Ignavibacterium album*, and *Arsenophonus nasoniae* were enriched in the BV group. We observed that 10 microbes at the species level were significantly different between the VVC and VVC_BV groups (LDA >3; Fig. 2J). *Prevotella scopos*, *Prevotella denticola*, *Prevotella dentalis*, *Porphyromonas asaccharolytica*, *Prevotella oris*, *Megasphaera elsdenii*, *Porphyromonas gingivalis*, *Ascaris suum*, *Phocaeicola dorei*, and *Butyricimonas faecalis* were greater in the VVC_BV group. Results showed that the structure of vaginal flora in the VVC_BV group was similar to that of the BV group.

### Significant differences in vaginal flora functional potentials between healthy women and patients with vaginitis

**Fig 3:**
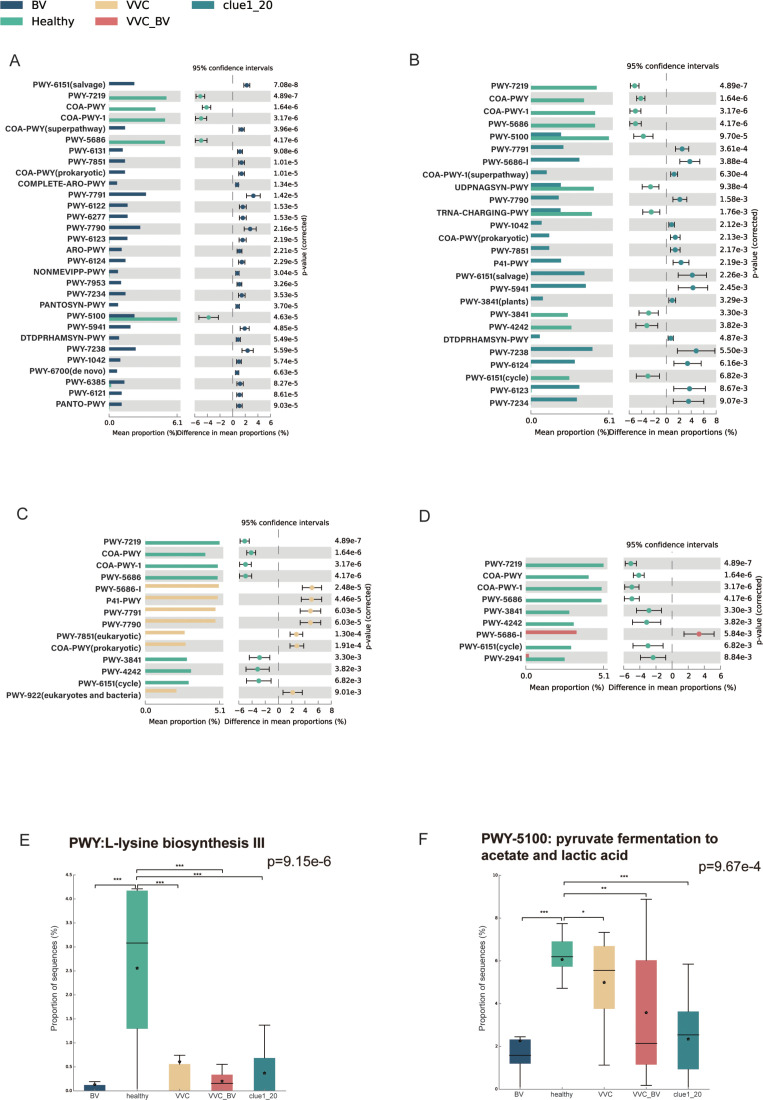
The comparisons of the vaginal microbiome functional potential among the five groups. (**A**) The different metabolic pathways between the healthy and BV groups (*P* < 0.001). (**B**) The different metabolic pathways between the healthy and Clue1_20 groups (*P* < 0.01). (**C**) The different metabolic pathways between the healthy and VVC groups (*P* < 0.01). (**D**) The different metabolic pathways between the healthy and VVC_BV groups (*P* < 0.01). (**E**) The comparison of the activity of L-lysine biosynthesis III among the five groups. (**F**) The comparison of the activity of pyruvate fermentation to acetate and lactic acid among the five groups. ^ns^*P*-value >0.05, ^*^P-value <0.05; ^**^P-value <0.01; ^***^P-value < 0.001 in panels E and F.

The microbial metabolic pathways were compared between the healthy group and other disease groups. Notably, in the BV group, pathways related to carbohydrate synthesis, such as the superpathway of coenzyme A biosynthesis III (mammals), chorismate biosynthesis I, sucrose biosynthesis II, and UMP biosynthesis III, as well as inosine-5′-phosphate biosynthesis I and III, were increased, while adenosine ribonucleotides *de novo* biosynthesis, coenzyme A biosynthesis II (mammals), and pyruvate fermentation to acetate and lactate II were decreased (*P* < 0.001; Fig. 3A). In the Clue1_20 group, pyruvate fermentation to acetate and lactate II, tRNA charging, and UDP-N-acetyl-D-glucosamine biosynthesis I were lower, while S-adenosyl-L-methionine salvage I and UMP biosynthesis III were higher compared to that in the healthy group (*P* < 0.01; Fig. 3B). In the VVC group, several pathways related to fungi were enriched, including coenzyme A biosynthesis II (eukaryotic) and mevalonate pathway I (eukaryotes and bacteria) (*P* < 0.01; Fig. 3C). Other enriched pathways in the VVC group were similar to those enriched in the BV group, such as UMP biosynthesis II and III (*P* < 0.01; Fig. 3C). The VVC_BV group was also enriched in pathways related to fungi, including coenzyme A biosynthesis II (eukaryotic) and coenzyme A biosynthesis I (prokaryotic); however, L-lysine biosynthesis II and S-adenosyl-L-methionine cycle I were decreased (*P* < 0.01; Fig. 3D). In conclusion, the potential functions of the vaginal flora in the Clue1_20 group were closer to those in the healthy than those of the BV group. Furthermore, not only bacteria-related pathways were predicted in the VVC_BV group, which were similar to those in the BV group, but also fungus-related pathways similar to those in the VVC group.

*Lactobacillus* provided an acidic environment for the vaginal environment of healthy women. The above analysis revealed significant enrichment of pathways related to pyruvate fermentation to acetate and lactate and amino acid biosynthesis in the healthy group. Thus, we compared these two pathways related to the potential function of *Lactobacillus* in five groups. As expected, they were significantly higher in the healthy group than BV, Clue1_20, VVC, and VVC_BV groups (*P* < 0.001, Fig. 3E; *P* < 0.001, *P* < 0.001, *P* < 0.05 and *P* < 0.01, respectively, Fig. 3F).

### Functional potential comparison of vaginal flora among patients with different types of vaginitis

**Fig 4:**
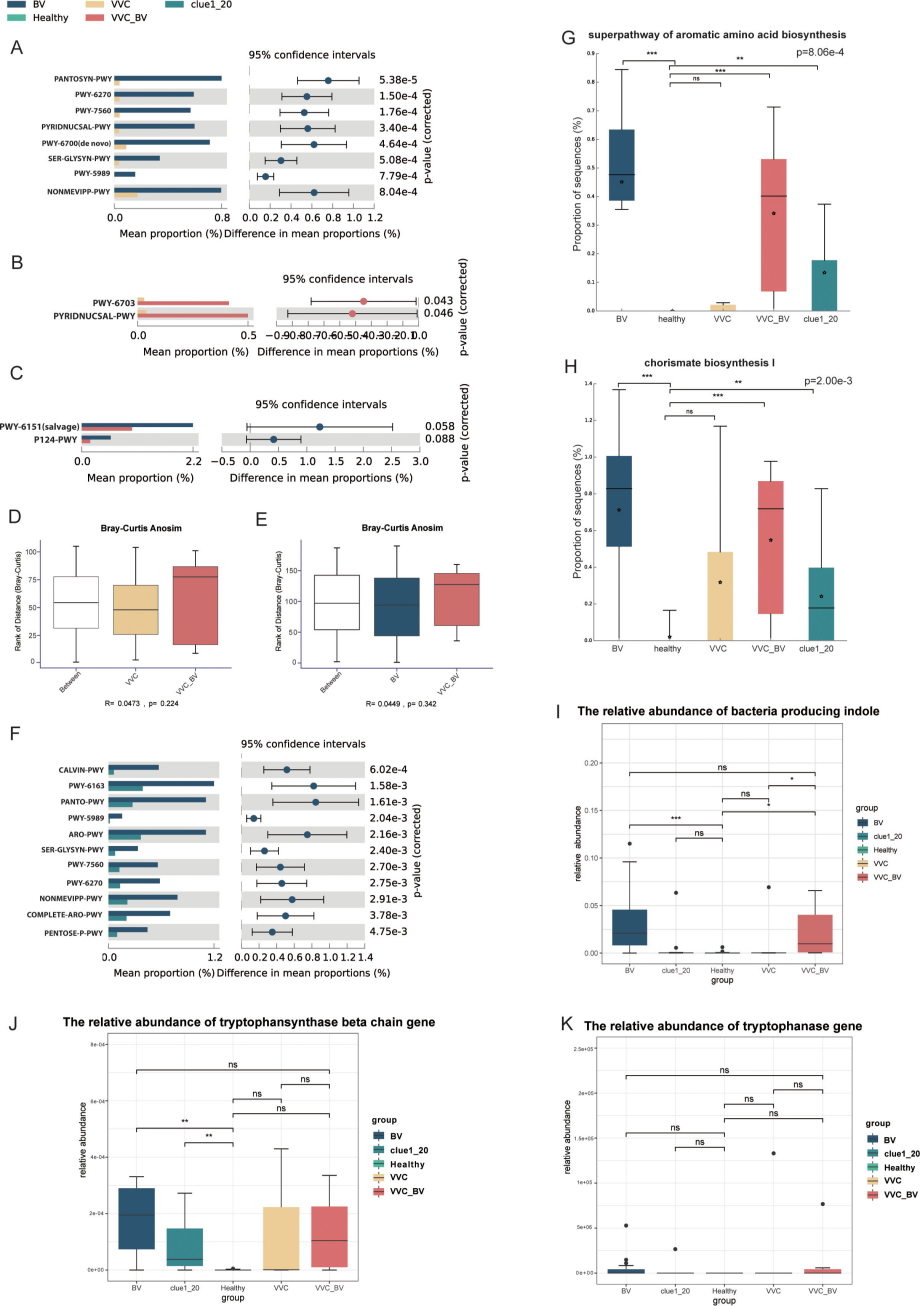
The differences of pathways of aromatic amino acid biosynthesis and related bacteria among the five groups and the comparison of the vaginal microbiome potential function among the BV, VVC, and VVC_BV groups. (**A**) The different metabolic pathways between the VVC and BV groups (*P* < 0.001). (**B**) The different metabolic pathways between the VVC and VVC_BV groups (*P* < 0.05). (**C**) The different metabolic pathways between the BV and VVC_BV groups (*P* < 0.01). (**D**) The correlation analysis for metabolomic data of the VVC and VVC_BV groups (Bray-Curtis Anosim, *R* = 0.0473, *P* = 0.224). (**E**) The correlation analysis for metabolomic data of the BV and VVC_BV groups (Bray-Curtis Anosim, *R* = 0.0449, *P* = 0.342). (**F**) The different metabolic pathways between the Clue1_20 and BV groups (*P* < 0.05). (**G**) The comparison of the relative abundance of the superpathway of aromatic amino acid biosynthesis among the five groups. (**H**) The comparison of the relative abundance of chorismite biosynthesis I (from 3-dehydroquinate) among the five groups. (**I**) The comparison of the relative abundance of bacteria producing indole among the five groups. (**J and K**) The comparisons of the relative abundance of tryptophansynthase beta chain (**J**) and tryptophanase (**K**) among the five groups. ^ns^*P*-value >0.05, ^*^P-value <0.05; ^**^P-value <0.01; ^***^P-value <0.001 in panels G, H, I, J, and K.

The BV group exhibited greater enrichment in certain bacterial pathways compared to the VVC group, including NAD *de novo* biosynthesis I (from aspartate), queuosine biosynthesis (*de novo*), and methylerythritol phosphate pathway I and II (*P* < 0.001; Fig. 4A). The main differences in bacterial pathways between the VVC and VVC_BV groups were NAD *de novo* biosynthesis I (from aspartate) and preQ_0_ biosynthesis (*P* < 0.05; Fig. 4B). Furthermore, the BV and VVC_BV groups showed marked differences in the *Bifidobacterium* shunt and S-adenosyl-L-methionine salvage I pathways, which are primarily rich in bacteria (*P* < 0.01; Fig. 4C). According to the Bray-Curtis Anosim analysis, the functional potential of the vaginal flora in the VVC_BV group was close to the VVC group (*R* = 0.0473, *P* = 0.224) and BV group (*R* = 0.0449, *P* = 0.342) (Fig. 4D and E).

### Relationship between aromatic amino acid biosynthesis and indole in the vagina of BV patients

Our study revealed significant enrichment of chorismate biosynthesis from 3-dehydroquinate and superpathway of aromatic amino acid biosynthesis in the BV group compared to the Clue1_20 group (*P* < 0.05; Fig. 4F). Further analysis using box plots across the five groups demonstrated that these pathways were the richest in the BV group, followed by the VVC_BV and Clue1_20 groups (*P* < 0.001, *P* < 0.001, and *P* < 0.01, respectively; Fig. 4G and H). Moreover, we examined the proportion of bacteria capable of producing indole and found that comparing to the healthy group, 25 bacterial counts were enriched in the BV group (*P* < 0.001), followed by the VVC_BV group (*P* < 0.001 and *P* < 0.05, respectively; Fig. 4I; Table S2). We conducted gene family-level analysis to assess the abundance of genes involved in tryptophan biosynthesis and metabolism, specifically the genes encoding tryptophansynthase β chain and tryptophanase. Results showed that the relative abundance of the encoding tryptophansynthase β chain gene was decreased in the healthy group and increased in the BV and Clue1_20 groups (*P* < 0.01; Fig. 4J and K). In conclusion, the BV group exhibited higher relative abundance in the aromatic amino acid biosynthesis pathway related to the abundance of BV-associated pathogens capable of producing indole compared to the Clue1_20 group.

### Probiotic properties of *L. crispatus* isolated from the vagina of healthy women

**Fig 5:**
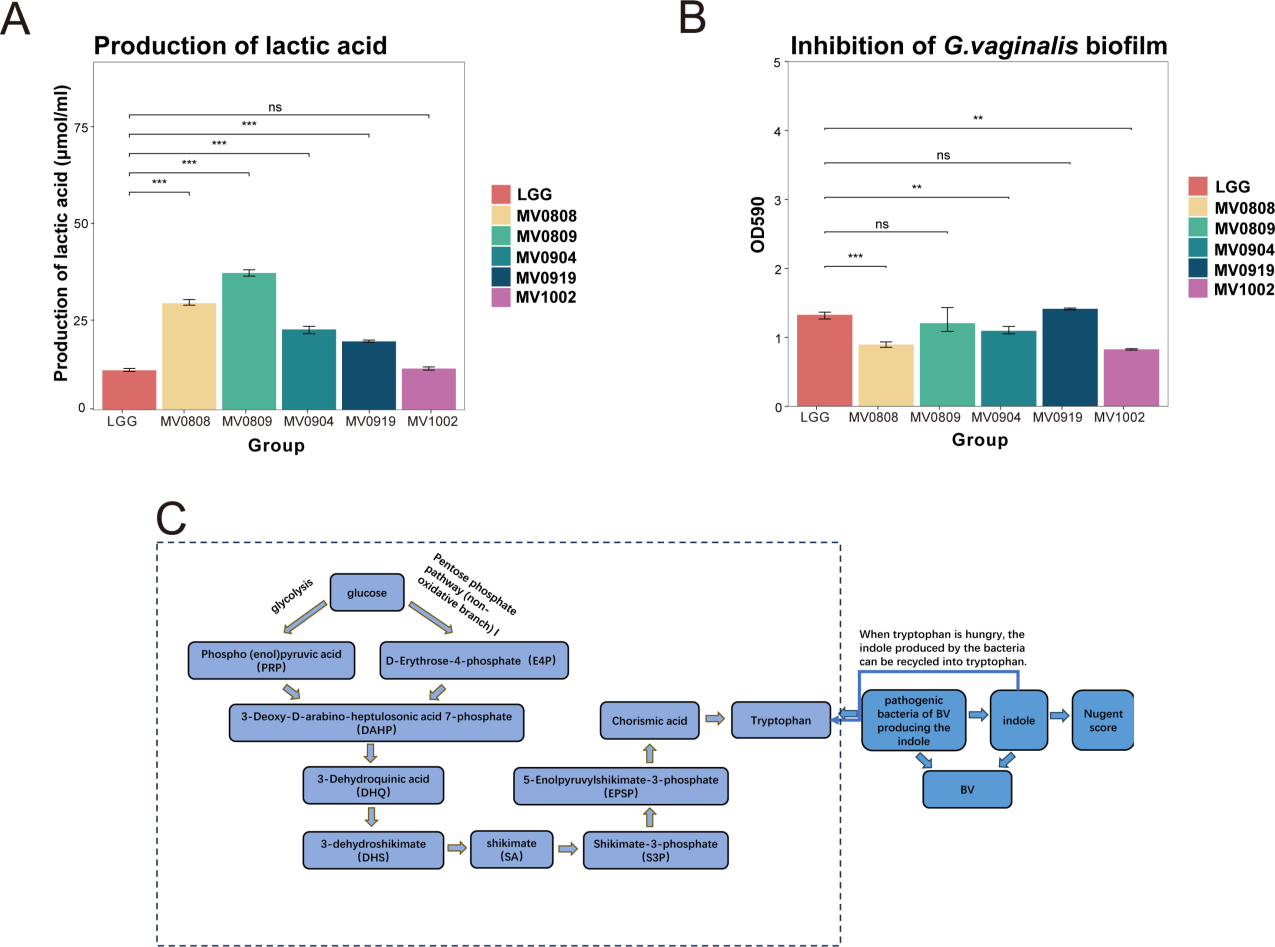
The probiotic properties of *L. crispatus* isolated from the vagina of healthy women and the aromatic amino acid synthesis pathways and indole metabolism in bacteria. (**A**) The capacity of *L. crispatus* in producing lactic acid among the five *L. crispatus* strains. (**B**) The capacity of *L. crispatus* in inhibiting *G. vaginalis* biofilms among the five *L. crispatus* strains. (**C**) The aromatic amino acid synthesis pathways and indole metabolism in bacteria. ^ns^*P*-value >0.05, ^*^P-value <0.05; ^**^P-value <0.01; ^**^P-value <0.001 in panels A and B.

Given the highest relative abundance of *L. crispatus* in the healthy group and the above findings on the possible effect of *Lactobacillus*, selective isolation of *L. crispatus* was performed to explore its possible role in the vaginal environment. Five strains of *L. crispatus* were isolated from vaginal samples taken from the healthy group. Their lactic acid production capacity was examined using a lactic acid content assay kit, with the international standard LGG (*Lactobacillus rhamnosus GG*) serving as a control. Results showed that MV0809 exhibited the highest lactic acid production ability, following by MV0808 (*P* < 0.001; Fig. 5A). Next, we evaluated the ability of *L. crispatus* to inhibit *G. vaginalis* biofilm formation. We measured the biofilm activity and formation of *G. vaginalis* (Fig. S3). The biofilms of *G. vaginalis* (CFU/mL) were cultured with the cell-free supernatant (CFS) of *L. crispatus* for 24 h. Results indicated that MV1002 (*P* < 0.01) and MV0808 (*P* < 0.001) exhibited greater inhibitory effects compared to the other strains (Fig. 5B). Thus, among the isolated strains, MV0808 demonstrated the best capacity in terms of producing lactic acid and inhibiting *G. vaginalis* biofilm formation.

## DISCUSSION

In our study, we employed metagenomic sequencing to characterize the biomarkers associated with various types of vaginitis. Our findings proposed transitional pattern hypothesis in the vaginal microbiota between healthy females, women with 1%–20% clue cells, and patients with BV. Furthermore, we characterized the vaginal microbiota relationships among patients with BV, VVC, and both BV and VVC. These findings expanded our understanding of the distinct characteristics of vaginal microbiota in females with vaginitis.

With the accumulation of research, our understanding of vaginal microecology in women continues to grow (27). In the current study, we conducted a comparative analysis between each disease group and the healthy group to gain an overview of the structural and potentially functional characteristics of the vaginal flora. In the healthy group, *L. crispatus* was the dominant bacteria in most samples, likely playing a significant role in the vaginal flora, but was decreased in the other groups (28). Compared to the healthy individuals, each disease group exhibited distinct bacterial biomarkers and individual functional pathways potentially. *G. vaginalis* was enriched in both the BV and Clue1_20 groups, although with a higher percentage in the former. Additionally, *Candida* was enriched in the VVC_BV and VVC groups, contributing to VVC infection (29). The high relative abundance of *C. albicans* in the VVC group was consistent with previous findings (30–32). However, *Candida* content was still lower in the VVC_BV group than in the VVC group. Interestingly, the VVC_BV group showed an increase in anaerobic bacteria, such as *G. vaginalis* and *Prevotella*, which were clinically considered the main pathogens of BV (33, 34). In the functional prediction of the flora, we found that certain pathways probably associated with *Lactobacillus*, including lactic acid and amino acid biosynthesis, were significantly enriched in the healthy group. Studies have indicated that healthy women with rich *Lactobacillus* in the vagina possess higher levels of amino acids in the vagina compared to patients with HPV or BV (35, 36). Particularly, ε-poly-L-lysine can inhibit the biofilm formation of *G. vaginalis* (37, 38). Moreover, women with gynecological diseases tend to exhibit a higher vaginal pH than healthy women due to the lower lactic acid in the vagina (pH 4.5) (39–42). Therefore, we selected two representative pathways, L-lysine biosynthesis III and pyruvate fermentation to acetate and lactic acid, for comparison in five groups and discovered that they were increased in the healthy group but decreased in other disease groups. Our finding was consistent with the report that pathways related to amino acid biosynthesis were lower in BV patients than in healthy women (36). In addition, the coenzyme A biosynthesis II (eukaryotic) pathway, associated with eukaryotic processes, was found to be enriched in both the VVC_BV and VVC groups. In conclusion, the enrichment of specific pathways in each group was closely related to the composition of their respective microbial species. The potential microbial function in the Clue1_20 group was close to the BV group. Moreover, the functional prediction results in the VVC_BV group exhibited similarities to those observed in the BV and VVC groups. These findings provided valuable insights into the distinct microbial and metabolic patterns associated with different vaginitis conditions.

We next explored the relationship between the presence of 1%–20% clue cells in the vagina and BV. Traditionally, the clinical diagnosis of BV is determined by vaginal discharge and odor, vaginal pH, and the proportion of clue cells (>20%), identified via microscopic examination (7, 43, 44). Clue cells are vaginal epithelial cells that show a presence of adherent bacteria, such as *G. vaginalis* (45). Some women with 1%–20% clue cells in the vagina experience symptoms similar to those of patients with BV (46). However, few studies have explored the condition of 1%–20% clue cells in women (47). In contrast to previous research, we included vaginal samples with 1%–20% clue cells in the study to explore differences compared to the BV and healthy groups. Our results hypothesized *Prevotella* as the most significantly different bacteria between the BV and Clue1_20 groups, showing an increase in the BV group and a decrease in the Clue1_20 group. A total of 14 species of *Prevotella* were present in our samples, with *P. intermedia* being the most abundant in the BV group. The conclusion of our study was consistent with the fact that *P. intermedia* had been reported as a potential risk factor for BV in women (48). Additionally, *Lactobacillus* was found to be the most abundant bacteria in the healthy group but was found at the lowest abundance in the BV group. Notably, the abundance of *Lactobacillus* in the Clue1_20 group was closer to that in the healthy group. *L. iners* is the dominant bacterium of community state type (CST-III) in the major vaginal community state types in women (49). However, previous studies have shown that *L. iners* may not show the same beneficial effects as other *Lactobacillus* from the vagina such as *L. crispatus* (50, 51). In this study, we found that although there was no significance on the relative abundance of *L. iners* in the three groups, *L. iners* was the predominant *Lactobacillus* in the Clue1_20 and BV groups. Based on these findings, we postulated that the vaginal condition with 1%–20% clue cells represented a transitional state between the healthy and BV groups, characterized by an abundance of *L. iners* and *G. vaginalis*, but lower content of *Prevotella* than BV.

Our study also showed that chorismate biosynthesis and superpathway of aromatic amino acid biosynthesis, known as the shikimic acid pathway in bacteria, were significantly enriched in the BV and VVC_BV groups. Chorismate biosynthesis constitutes that part of the shikimic acid pathway that facilitates the synthesis of tryptophan in bacteria (Fig. 5C) (52). Tryptophan is a known aromatic amino acid present in bacteria (53, 54). Due to the presence of the tryptophanase enzyme, some species of vaginal pathogens such as *Prevotella*, *Chlamydia*, and certain bacteria associated with BV possess the ability to degrade tryptophan into indole (55–59). Moreover, the levels of indole in the vaginal environment of BV patients are significantly higher than those in healthy individuals (59). Thus, we first assessed the abundance of indole-producing bacteria in the collected samples in accordance with the research of Noa et al. (55), and the result was in line with the above reports. Notably, the relative abundances of these bacteria were significantly higher in the BV group, followed by the VVC_BV group, which contained more bacteria associated with BV. Then, we observed higher abundance of the gene encoding tryptophansynthase in the BV group than in other groups. In summary, we speculated that there were more indole-producing bacteria in the vagina of patients with BV, which might be related to the disease progression.

In addition, we conducted in-depth comparisons of the vaginal flora among BV, VVC, and mixed (VVC_BV) infections. Previous studies have suggested that *Candida* can produce metabolites with the ability to inhibit bacterial growth, although there is no evidence to support the notion that it selectively inhibits only Gram-negative or Gram-positive bacteria, such as *Lactobacillus* (33, 60). Our analysis of the comparison between the BV and VVC groups showed the related result that the presence of *Lactobacillus* in the vaginal flora of VVC patients was markedly higher than that in BV patients. Therefore, we speculated that *Candida* might not inhibit the growth of *Lactobacillus*, which was consistent with the conclusions of Macklaim et al. (33, 61). In addition, our study showed that the vaginal flora of the VVC_BV group was also higher in *G. vaginalis*, which was similar to the situation of patients with BV. In functional prediction, we also found that most of the pathways enriched in VVC_BV group, such as coenzyme A biosynthesis pathway related to fatty acid synthesis, were associated with BV disease progression (62, 63). Previous report showed that the high concentrations of fatty acids in the vagina could trigger elevated levels of inflammatory cytokines (64). All in all, the above results indicate that the infection of BV combined with VVC was complicated.

According to the above study and the previous reports, we observed that *L. crispatus* was the dominant bacteria in the vagina of healthy women (65). As a result, we isolated and confirmed the positive effects of *Lactobacillus* in the vaginal flora based on *in vitro* experiments. Due to the important role of lactic acid in the vaginal environment, we first tested the ability of *L. crispatus* strains to produce lactic acid. Additionally, colonization of *G. vaginalis* in the vagina of BV patients has been shown to correlate with its biofilm formation (66, 67). Current studies indicated that *Lactobacillus* could inhibit the biofilm formation of *G. vaginalis* (68, 69). Then basing on this, we investigated the inhibitory capacity of *L. crispatus* strains isolated from healthy women against *G. vaginalis* biofilm formation and lactate production *in vitro*. Previous studies have shown that *Lactobacillus rhamnosus* has a good effect on enhancing the restoration of the vaginal flora of BV patients. Besides, it has the capacity to inhibit the biofilm formation of pathogenic bacteria such as *G. vaginalis* (70–72). Therefore, it was used as the control strain in our study. Our findings indicated that the MV0808 strain showed considerable potential as a probiotic candidate. As such, this strain would be the focus of future research endeavors, offering exciting possibilities for the development of targeted probiotic interventions in vaginitis management.

However, we did not conduct metabolomic analysis on the samples due to the high requirements for vaginal secretion content. As a result, we were unable to confirm whether the abundance of indole in the BV group was higher than that in other groups. Metabolome analysis could better support the conjecture regarding the relationship between indole and the transition state of the healthy and BV, which was a limitation of this study.

In conclusion, our study provided valuable insights into the characteristics of the vaginal flora in different types of vaginitis. We found that the presence of 1%–20% clue cells in the vagina might be a transitional state between a healthy flora and BV. Next, we would conduct the prospective study with repeated sampling of volunteers and found that the co-infected patients exhibited a flora composition more likely to that of VVC, and their potentially functional pathways showed similarities to BV and VVC. This observation highlights the complexity of mixed infections and may have clinical implications for treatment strategies. Furthermore, considering the importance of *L. crispatus* in promoting vaginal health, we isolated multiple strains from vaginal samples of healthy individuals and selected the most promising strain with potential probiotic benefits. Our future research will focus on evaluating the antibacterial efficacy of *L. crispatus* against indole-producing pathogens for developing targeted therapeutic interventions.

## MATERIALS AND METHODS

### Human subjects

The study was cross-sectional and approved by the Ethics Committee of the West China Second University Hospital of Sichuan University (2022-054) and strictly adhered to the principles of the Declaration of Helsinki. Each volunteer provided signed informed consent. All recruited volunteers were patients and healthy females at the Gynecological Outpatient Clinic of West China Second University Hospital from China, and each female underwent vaginal microecological examination including cleanliness, clue cell content, pH, hydrogen peroxide concentration, sialidase (SNa), leucocyte esterase, and the presence of budding yeast cells with or without pseudohyphae and trichomonas. The criteria for inclusion in the study were as follows: (i) aged between 24 and 55 years, (ii) a history of sexual activity, (iii) a regular menstrual cycle, (iv) no use of antibiotics or estrogens in the previous 30 days, (v) no use of vaginal medication or lavage in the previous 7 days, and (vi) no cervix-related surgeries in the previous 3 months. The 49 samples were divided into five groups: healthy (*N* = 8), BV (*N* = 15), Clue1_20 (*N* = 11), VVC (*N* = 10), and VVC_BV (*N* = 5).

### Sample collection

Vaginal secretions were collected using sterile medical cotton swabs, which were then placed in sterile tubes and frozen at −80°C. BV was diagnosed when the number of clue cells exceeded 20, pH ≥4.5, and the amine test was positive (7, 73). Likewise, VVC was diagnosed based on the presence of budding yeast cells and pseudohyphae (74). In addition to the inclusion criteria, healthy volunteers should also meet the following: (i) no history of gynecological diseases and urinary tract infections in the previous three months; (ii) a normal vaginal microbiome test; (iii) a negative HPV test; and (iv) no clue cells, budding yeast cells, and pseudohyphae in the vagina.

### DNA extraction and metagenomic processing

Extract the total DNA according to the TIANNamp Bacteria DNA Kit (Tiangen Biotech Co., Ltd., China) instructions and measure DNA concentration and quality by the NanoDrop. The Covaris ultrasonic crusher was used to randomly break DNA into less than 500-bp fragments. After constructing the library, the detection of library mass and concentration were performed, respectively, by Qubit 2.0. The integrity of the DNA fragment and the size of the insert were measured by AATI (Advanced Analytical Technologies) and quantified by QPCR (Quantitative Real-time PCR). The concentration of the final library was >5 ng/µL in a volume of 50 µL. The qualified DNA library was sequenced bilaterally on the Illumina NovoSeq 6000 platform (Novogene Co., Ltd., China). Trimmomatic was used to remove low-quality reads. Based on Bowtie2 (version 2.4.2), sequences coming from the human genome were removed. Raw data have been submitted to the China National GeneBank DataBase (https://db.cngb.org/) with the accession number CNP0004576. Samples of the healthy group were downloaded from the National Center for Biotechnology Information Sequence Read Achieve (http://www.ncbi.nlm.nih.gov/sra) with the accession number CNP0004123, which are the samples in the paper to be published in our laboratory. The DNA extraction and sequencing methods for the healthy samples are the same as those for the disease group.

### Gene assembly and function prediction

Assembly analysis was performed by MEGAHIT (version 1.2.9) with the option “--min-contig-len 300” (75). Prodigal (version 3.0.2) was used to obtain gene prediction results with the option “-p meta.” In addition, CD-HIT (version 4.5.6) was used with default parameters (identity 95%; coverage 90%) to cluster and to remove redundant sequences, thus obtaining non-redundant gene set sequences (unigenes). Salmon (version 1.3.0) was used to compare clean reads to unigenes to obtain information on the relative abundance of genes with the option “--meta.” The annotation results obtained by database comparison were corresponded to the abundance information table of each gene, and the abundance information corresponding to the gene annotation results was obtained. Anatomy of the potential functional content of QC sequences (UniRef90 gene family and MetaCyc metabolic pathway) (76). The abundance of gene families and microbial metabolic pathways were assessed by HUMAnN3 using MetaCyc and UniRef90 EC filtering databases (77) and normalized by copies per million. Default parameters were used for software without specified parameters. Besides, STAMP (version 2.1.3) was used to predict the different functional pathways among the five groups with ANOVA (Analysis of Variance) test.

### Identification of taxa in metagenomes

Kraken2 (version 2.1.2) and Bracken (version 2.5.0) were used for species taxonomic annotation (78). The LEfSe was used to perform differential analysis of taxa. In addition, in the LEfSe analysis, *P* < 0.05 and LDA >2 obtained by linear discriminant analysis were considered statistically significant. PCoA was used to evaluate the similarities or differences in the composition of the sample communities based on Bray-Curtis distances by adonis (79). The statistical analysis was performed by R (version 4.2.3) using vegan (version 2.6-4) and ggplot2 (version 3.4.4) packages, and the *P* value was calculated by Wilcoxon’s rank-sum test with *P* < 0.05.

### Microbial strains and growth conditions

*Lactobacillus crispatus* strains were isolated from the vaginal secretion of healthy females collected in the West China Second University Hospital. They were grown anaerobically in MRS *(*DeMan, Rogosa and Sharpe medium) broth for 24 h (37°C, CO_2_) as well as *Lactobacillus rhamnosus* strain (LGG) (ATCC 53103). *Gardnerella vaginalis* strain (ATCC 49145) was cultured in NYC III broth under anaerobic conditions for 24 h (37 ℃, CO_2_).

### Capacity to produce the lactic acid

*L. crispatus* (1 × 10^6^ CFU/mL) in MRS broth were centrifuged (10,000 × *g*, 10 min, 4°C) and left for the supernatant. Filter the supernatant through 0.22-µm filter membrane to obtain the CFS. Then, follow the lactic acid content assay kit (Shanghai Yuanye Bio-Technology Co., Ltd). LGG was the standard strain serving as the control. Results were the mean ± SEM from three independent experiments (each with *n* = 3).

### Capacity to inhibit the biofilm of *G. vaginalis*

A 100-µL *G. vaginalis* (1 × 10^8^ CFU/mL) was incubated in each cell of a 96-well microplate for 24 h (37°C, CO_2_) to form the biofilm. Crystal violet (CV) staining (Guangdong Huankai Microbial Sci. & Tech. Co., Ltd.) was used to evaluate the viability of *G. vaginalis* biofilm at incubation for 8, 16, 18, and 24 h. The biofilm incubated for 24 h was selected for the following experiment. Add 100-µL NYC III broth and 100-µL CFS of *L. crispatus* (1 × 10^6^ CFU/mL) to the cells with *G. vaginalis* biofilm and incubate them for 24 h. CV staining was used to examine the candidate strains’ capacity. Discard the liquid and gently wash the cell with phosphate-buffered saline (PBS) three times; add 100-µL methanol to fix the cells for 15 min. Discard methanol, air dry, add 100-µL 0.5% (wt/vol) CV, and stain for 20 min. Wash with PBS three times, discard 0.5% CV, and air dry. Add 100-µL 33% (vol/vol) acetic acid for 30 min. The optical density (OD) at 590 nm was measured by the microplate [Thermo Fisher Scientific (China-HK) Holding Co., Ltd.] (72). LGG was the standard strain serving as the control. Results were the mean ± SEM from three independent experiments (each with *n* = 3).

### STORMS Checklist

This study has been completed according to the STORMS Checklist (DOI: 10.5281/zenodo.10065440).

## SUPPLEMENTAL MATERIAL

Fig. S1 msystems.01377-23-s0001.pdf Top 20 most abundant bacteria in the BV, Clue1_20, and healthy group at the family level.

Fig. S2 msystems.01377-23-s0002.pdf The average proportion of host data of the five groups in this study.

Fig. S3 msystems.01377-23-s0003.pdf The biofilm formation activity of *G. vaginalis*.

Table S1 msystems.01377-23-s0004.pdf Quality control data of metagenomic sequencing for all samples in this study.

Table S2 msystems.01377-23-s0005.pdf Indole-producing bacteria in this study.

Table S3 msystems.01377-23-s0006.pdf The proportion of human DNA of each sample in this study.

Table S4 msystems.01377-23-s0007.pdf The average number of non-host reads sequenced per sample in this study.

Table S5 msystems.01377-23-s0008.pdf Detailed description of the potential functional pathway.

ASM does not own the copyrights to Supplemental Material that may be linked to, or accessed through, an article. The authors have granted ASM a non-exclusive, world-wide license to publish the Supplemental Material files. Please contact the corresponding author directly for reuse.
