# Supplementary material for: Interpretation of vaginal metagenomic characteristics in different types of vaginitis
Source: mSystems. 2024 Feb 16;9(3):e01377-23. doi: 10.1128/msystems.01377-23 (PMC10949516; doi:10.1128/msystems.01377-23)
Supplement: Fig. S1 — Top 20 most abundant bacteria in the BV, Clue1_20, and healthy group at the family level. [file msystems.01377-23-s0001.pdf]

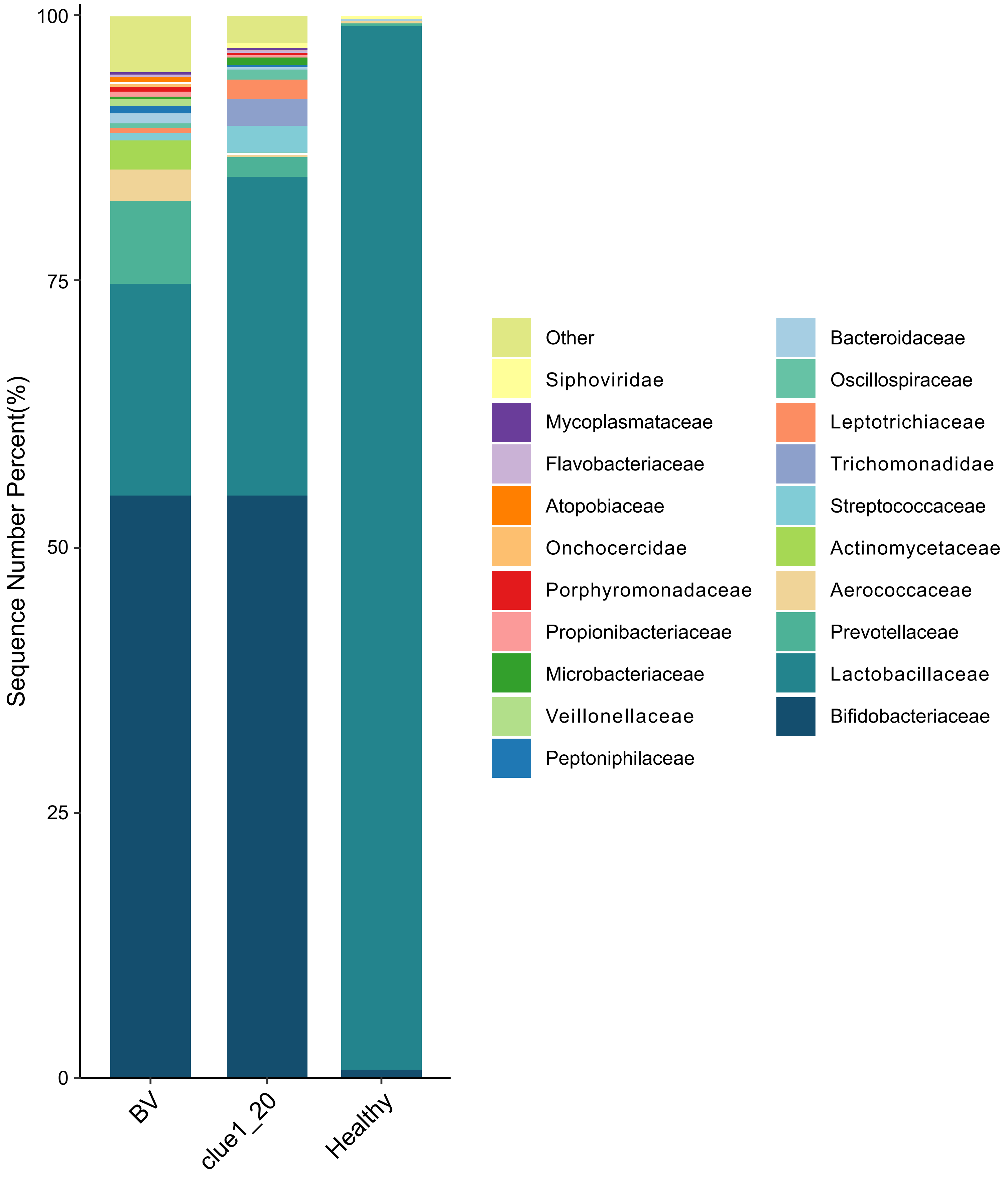


**Fig. S1. Top 20 most abundant bacteria in the BV, Clue1_20 and healthy group at the family level.** The composition of taxa in the BV, Clue1_20 and healthy groups was compared at the family level.
