# Supplementary material for: Interpretation of vaginal metagenomic characteristics in different types of vaginitis
Source: mSystems. 2024 Feb 16;9(3):e01377-23. doi: 10.1128/msystems.01377-23 (PMC10949516; doi:10.1128/msystems.01377-23)
Supplement: Fig. S2 — The average proportion of host data of the five groups in this study. [file msystems.01377-23-s0002.pdf]

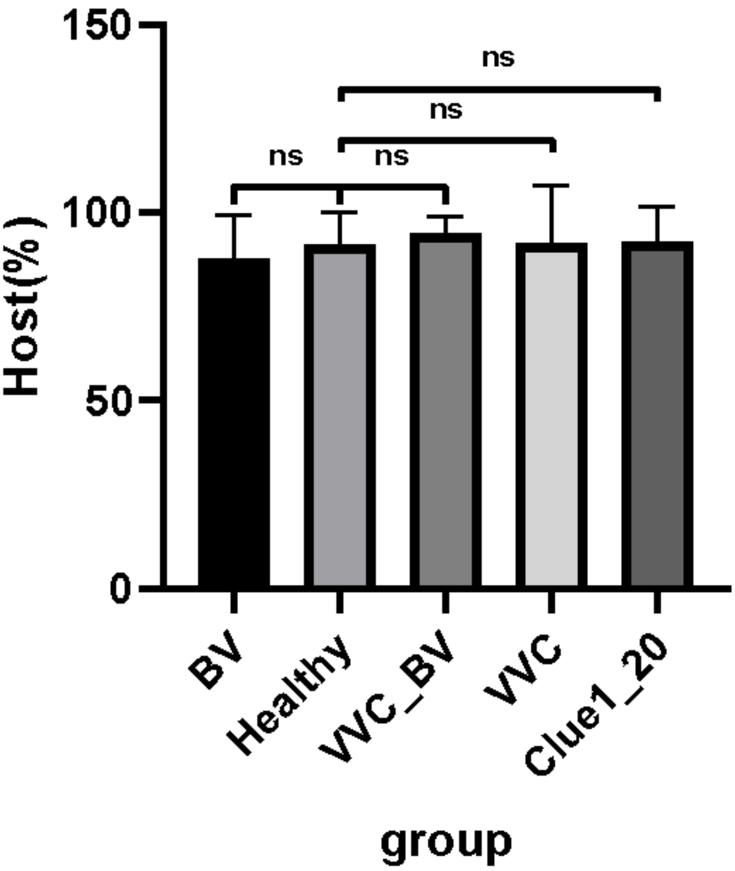

Fig. S2. The average proportion of host data of the five groups in this study. The average percents of human DNA (%) of the samples of the five groups were analyzed by GraphPad Prism version 8.0.1with ANOVA test (^{ns}, $P > 0.05$).
