# Supplementary material for: Interpretation of vaginal metagenomic characteristics in different types of vaginitis
Source: mSystems. 2024 Feb 16;9(3):e01377-23. doi: 10.1128/msystems.01377-23 (PMC10949516; doi:10.1128/msystems.01377-23)
Supplement: Fig. S3 — The biofilm formation activity of G. vaginalis. [file msystems.01377-23-s0003.pdf]

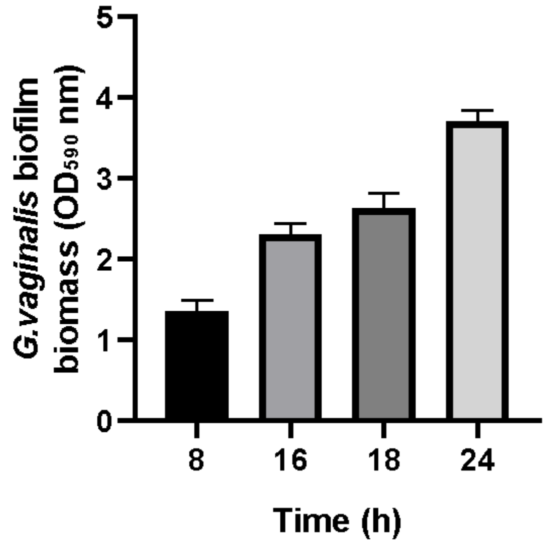

Fig. S3. The biofilm formation activity of *G. vaginalis*. *G. vaginalis* (10^8 CFU/mL) biofilm formation after incubating for 8, 16, 18 and 24 h. The biofilm biomass was measured by crystal violet (CV) staining (OD_{590} nm) and analyzed by GraphPad Prism version 8.0.1. Results were the mean ± SEM from 3 independent experiments (each with n = 3).
