# Supplementary material for: Interpretation of vaginal metagenomic characteristics in different types of vaginitis
Source: mSystems. 2024 Feb 16;9(3):e01377-23. doi: 10.1128/msystems.01377-23 (PMC10949516; doi:10.1128/msystems.01377-23)
Supplement: Table S1 — Quality control data of metagenomic sequencing for all samples in this study. [file msystems.01377-23-s0004.pdf]

Table S1. Quality control data of metagenomic sequencing for all samples in this study.

Sample	Library Name	Raw Reads	Clean Reads	Raw Base(G)	Clean Base(G)	Effective(%)	Error(%)	Q20(%)	Q30(%)	GC(%)
VVC_BV003	FDSW230182099-1r	33594776	33324184	5039216400	4998627600	99.19	0.03	96.79	91.91	41.2
BV032	FDSW230182077-1r	33647376	33452940	5047106400	5017941000	99.42	0.03	97.04	92.31	42.62
VVC_BV001	FDSW230182097-1r	33565546	33352328	5034831900	5002849200	99.36	0.03	96.74	91.84	44.35
BV034	FDSW230182079-1r	35505044	35319148	5325756600	5297872200	99.48	0.03	96.84	91.94	42.56
VVC_BV002	FDSW230182098-1r	33165712	32799134	4974856800	4919870100	98.89	0.03	96.36	91.37	47.57
BV023	FDSW230182068-1r	33997144	33702964	5099571600	5055444600	99.13	0.03	96.28	90.77	42.04
BV033	FDSW230182078-1r	35949584	35775142	5392437600	5366271300	99.51	0.03	96.46	91.1	41.01
VVC008	FDSW230182089-1r	36270624	35883598	5440593600	5382539700	98.93	0.03	96.49	91.36	43.87
BV029	FDSW230182074-1r	35571028	35320468	5335654200	5298070200	99.3	0.03	96.66	91.61	42.43
VVC001	FDSW230182082-1r	35526484	35384296	5328972600	5307644400	99.6	0.03	96.99	92.2	41.76
BV036	FDSW230182081-1r	33687508	33489280	5053126200	5023392000	99.41	0.03	96.83	91.98	41.48
BV030	FDSW230182075-1r	37407204	37163266	5611080600	5574489900	99.35	0.03	97.11	92.46	40.74
VVC_BV004	FDSW230182100-1r	37454138	37225402	5618120700	5583810300	99.39	0.03	96.97	92.21	41.06
VVC003	FDSW230182084-1r	37232872	37036646	5584930800	5555496900	99.47	0.03	96.74	91.75	42.43
VVC_BV005	FDSW230182101-1r	36024802	35843344	5403720300	5376501600	99.5	0.03	96.51	91.22	41.33
VVC005	FDSW230182086-1r	38850702	38601128	5827605300	5790169200	99.36	0.03	96.8	91.95	43.42
VVC004	FDSW230182085-1r	34909190	34727250	5236378500	5209087500	99.48	0.03	96.94	92.21	42.07
VVC002	FDSW230182083-1r	35537994	35138224	5330699100	5270733600	98.88	0.03	96.9	92.24	43.54
BV031	FDSW230182076-1r	35632436	35469484	5344865400	5320422600	99.54	0.03	96.74	91.71	41.19
BV008	FDSW230182062-1r	36311016	36110180	5446652400	5416527000	99.45	0.03	96.61	91.52	42.91
BV001	FDSW230182055-1r	39144972	38905276	5871745800	5835791400	99.39	0.03	96.37	90.96	41.55
BV002	FDSW230182056-1r	38071428	36773616	5710714200	5516042400	96.59	0.03	96.88	92.08	42.64
BV009	FDSW230182063-1r	38646942	38264686	5797041300	5739702900	99.01	0.03	96.88	92.09	41.95
BV025	FDSW230182070-1r	37633054	37065852	5644958100	5559877800	98.49	0.03	96.38	91.22	43.82

BV006	FDSW230182060-1r	38465332	38229262	5769799800	5734389300	99.39	0.03	96.75	91.8	43.09
BV021	FDSW230182066-1r	37499262	36794446	5624889300	5519166900	98.12	0.03	96.57	91.44	42.44
BV003	FDSW230182057-1r	33450854	33229946	5017628100	4984491900	99.34	0.03	97.15	92.68	42.53
BV007	FDSW230182061-1r	39232678	38297354	5884901700	5744603100	97.62	0.03	96.81	91.97	42.03
BV005	FDSW230182059-1r	36783560	36511380	5517534000	5476707000	99.26	0.03	96.54	91.3	42.33
BV019	FDSW230182065-1r	33146012	32871590	4971901800	4930738500	99.17	0.03	96.98	92.24	41.31
BV024	FDSW230182069-1r	38055304	37251692	5708295600	5587753800	97.89	0.03	96.94	92.31	42.81
BV004	FDSW230182058-1r	37572394	37315070	5635859100	5597260500	99.32	0.03	96.52	91.3	42.26
BV014	FDSW230182064-1r	39176294	38946282	5876444100	5841942300	99.41	0.03	96.99	92.28	41.69
VVC006	FDSW230182087-2r	34694006	27027616	5204100900	4054142400	77.9	0.04	91.41	85.58	41.03
BV028	FDSW230182073-2r	33786796	33428782	5068019400	5014317300	98.94	0.03	96.87	91.92	41.9
BV027	FDSW230182072-2r	34280778	24757844	5142116700	3713676600	72.22	0.04	90.83	85.07	41.75
VVC010	FDME230182091-1a	48920274	48589100	7338041100	7288365000	99.32	0.03	97.05	92.02	40.65
VVC009	FDME230182090-1a	46009932	45251846	6901489800	6787776900	98.35	0.03	96.54	91.02	41.29
BV026	FDME230182071-1a	33175758	32770574	4976363700	4915586100	98.78	0.03	96.15	90.04	40.39
BV035	FDME230182080-1a	47702074	47166858	7155311100	7075028700	98.88	0.03	96.86	91.91	43.07
VVC007	FDME230182088-1r	35132702	34817564	5269905300	5222634600	99.1	0.03	97.68	93.55	40.09
