# Supplementary material for: Interpretation of vaginal metagenomic characteristics in different types of vaginitis
Source: mSystems. 2024 Feb 16;9(3):e01377-23. doi: 10.1128/msystems.01377-23 (PMC10949516; doi:10.1128/msystems.01377-23)
Supplement: Table S2 — Indole-producing bacteria in this study. [file msystems.01377-23-s0005.pdf]

Table S2. Indole-producing bacteria in this study.

Species	Indole	Species	Indole
Clostridium novyi	+	Vibrio tapetis	+
Clostridium tetani	+	Vibrio vulnificus	+
Klebsiella oxytoca	+	Yersinia enterocolitica	+
Morganella morganii	+	Yersinia frederiksenii	+
Proteus vulgaris	+	Haemophilus influenzae	+
Providencia alcalifaciens	+	Escherichia coli	+
Treponema denticola	+	Porphyromonas asaccharolytica	+
Vibrio harveyi	+	Fusobacterium nucleatum	+
Vibrio mediterranei	+	Prevotella intermedia	+
Vibrio parahaemolyticus	+	Porphyromonas gingivalis	+
Enterococcus faecalis	+		
