# Supplementary material for: Interpretation of vaginal metagenomic characteristics in different types of vaginitis
Source: mSystems. 2024 Feb 16;9(3):e01377-23. doi: 10.1128/msystems.01377-23 (PMC10949516; doi:10.1128/msystems.01377-23)
Supplement: Table S3 — The proportion of human DNA of each sample in this study. [file msystems.01377-23-s0006.pdf]

Table S3. The proportion of human DNA of each sample in this study.

Sample	group	Filtered (%)	Host (%)	Sample	group	Filtered (%)	Host (%)
BV002	BV	11.19	88.81	MV001	Healthy	3.95	96.05
BV005	BV	2.79	97.21	MV002	Healthy	2.04	97.96
BV006	BV	3.86	96.14	MV004	Healthy	26.67	73.33
BV007	BV	13.91	86.09	MV008	Healthy	12.43	87.57
BV008	BV	0.82	99.18	MV010	Healthy	4.67	95.33
BV009	BV	3.38	96.62	MV011	Healthy	2.41	97.59
BV019	BV	24.19	75.81	MV013	Healthy	3.88	96.12
BV021	BV	13.19	86.81	MV014	Healthy	11.81	88.19
BV024	BV	6.71	93.29	BV001	clue1_20	1.14	98.86
BV025	BV	7.68	92.32	BV003	clue1_20	4.92	95.08
BV026	BV	7.92	92.08	BV004	clue1_20	1.20	98.80
BV028	BV	2.60	97.40	BV014	clue1_20	6.92	93.08
BV030	BV	26.51	73.49	BV023	clue1_20	34.47	65.53
BV032	BV	44.85	55.15	BV027	clue1_20	7.78	92.22
BV034	BV	17.03	82.97	BV029	clue1_20	2.04	97.96
VVC_BV001	VVC_BV	12.51	87.49	BV031	clue1_20	11.14	88.86
VVC_BV002	VVC_BV	0.50	99.50	BV033	clue1_20	8.87	91.13
VVC_BV003	VVC_BV	7.47	92.53	BV035	clue1_20	3.44	96.56
VVC_BV004	VVC_BV	2.78	97.22	BV036	clue1_20	3.66	96.34
VVC_BV005	VVC_BV	4.79	95.21	VVC006	VVC	5.79	94.21
VVC001	VVC	5.35	94.65	VVC007	VVC	52.20	47.80
VVC002	VVC	0.93	99.07	VVC008	VVC	4.46	95.54
VVC003	VVC	3.17	96.83	VVC009	VVC	2.99	97.01
VVC004	VVC	2.21	97.79	VVC010	VVC	3.85	96.15
VVC005	VVC	1.22	98.78				
