# Supplementary material for: Interpretation of vaginal metagenomic characteristics in different types of vaginitis
Source: mSystems. 2024 Feb 16;9(3):e01377-23. doi: 10.1128/msystems.01377-23 (PMC10949516; doi:10.1128/msystems.01377-23)
Supplement: Table S4 — The average number of non-host reads sequenced per sample in this study. [file msystems.01377-23-s0007.pdf]

Table S4. The average number of non-host reads sequenced per sample in this study.

Sample	group	Reads Num	Sample	group	Reads Num
BV001	clue1_20	375364	BV036	clue1_20	1042860
BV002	BV	3639774	VVC001	Healthy	1670230
BV003	clue1_20	1435246	VVC002	Healthy	280944
BV004	clue1_20	384100	VVC003	Healthy	1031242
BV005	BV	889300	VVC004	Healthy	672072
BV006	BV	1286986	VVC005	Healthy	411432
BV007	BV	4637032	VVC006	Healthy	1399756
BV008	BV	258310	VVC007	Healthy	16862256
BV009	BV	1120818	VVC008	Healthy	1398740
BV014	clue1_20	2366360	VVC009	VVC_BV	1181408
BV019	BV	6975270	VVC010	VVC_BV	1665416
BV021	BV	4267774	VVC_BV001	VVC_BV	3662782
BV023	clue1_20	10146830	VVC_BV002	VVC_BV	135668
BV024	BV	2196528	VVC_BV003	VVC_BV	2178706
BV025	BV	2475692	VVC_BV004	VVC	916124
BV026	BV	2215734	VVC_BV005	VVC	1500424
BV027	clue1_20	1732476	MV001	VVC	2462668
BV028	BV	753360	MV002	VVC	1244566
BV029	clue1_20	618382	MV004	VVC	16819366
BV030	BV	8697418	MV008	VVC	7476436
BV031	clue1_20	3451408	MV010	VVC	3600728
BV032	BV	13440534	MV011	VVC	1526990
BV033	clue1_20	2767534	MV013	VVC	3068322
BV034	BV	5315304	MV014	VVC	7840934
BV035	clue1_20	1425848			
