# Supplementary material for: Interpretation of vaginal metagenomic characteristics in different types of vaginitis
Source: mSystems. 2024 Feb 16;9(3):e01377-23. doi: 10.1128/msystems.01377-23 (PMC10949516; doi:10.1128/msystems.01377-23)
Supplement: Table S5 — Detailed description of the potential functional pathway. [file msystems.01377-23-s0008.pdf]

Table S5. Detailed description of the potential functional pathway.

ID	Description
ARO-PWY	chorismate biosynthesis I
CALVIN-PWY	Calvin-Benson-Bassham cycle
COA-PWY	coenzyme A biosynthesis I
COA-PWY (prokaryotic)	coenzyme A biosynthesis I (prokaryotic)
COA-PWY-1	coenzyme A biosynthesis II (mammalian)
COA-PWY-1 (superpathway)	superpathway of coenzyme A biosynthesis III (mammals)
COMPLETE-ARO-PWY	superpathway of aromatic amino acid biosynthesis
DTDPRHAMSYN-PWY	dTDP-β -L-rhamnose biosynthesis
NONMEVIPP-PWY	methylerythritol phosphate pathway I
P124-PWY	Bifidobacterium shunt
P41-PWY	pyruvate fermentation to acetate and (S)-lactate I
PANTO-PWY	phosphopantothenate biosynthesis I
PANTOSYN-PWY	pantothenate and coenzyme A biosynthesis I
PENTOSE-P-PWY	pentose phosphate pathway
PWY-1042	glycolysis IV
PWY-2941	L-lysine biosynthesis II
PWY-3841	folate transformations II
PWY-3841 (plants)	folate transformations II (plants)
PWY-4242	pantothenate and coenzyme A biosynthesis III
PWY-5100	pyruvate fermentation to acetate and lactate II
PWY-5686	UMP biosynthesis
PWY-5686	UMP biosynthesis
PWY-5686-I	UMP biosynthesis I
PWY-5941	glycogen degradation II
PWY-5989	stearate biosynthesis II (bacteria and plants)
PWY-6121	5-aminoimidazole ribonucleotide biosynthesis I
PWY-6122	5-aminoimidazole ribonucleotide biosynthesis II
PWY-6123	inosine-5'-phosphate biosynthesis I
PWY-6124	inosine-5'-phosphate biosynthesis II
PWY-6151	S-adenosyl-L-methionine cycle I
PWY-6151 (cycle)	S-adenosyl-L-methionine cycle I
PWY-6151 (cycle)	S-adenosyl-L-methionine cycle I
PWY-6151 (salvage)	S-adenosyl-L-methionine salvage I
PWY-6163	chorismate biosynthesis from 3-dehydroquinate
PWY-6270	isoprene biosynthesis I
PWY-6277	superpathway of 5-aminoimidazole ribonucleotide
PWY-6385	peptidoglycan biosynthesis III (mycobacteria)
PWY-6700	queuosine biosynthesis
PWY-6700 (de novo)	queuosine biosynthesis I (de novo)
PWY-6703	preQ_0 biosynthesis
PWY-7219	adenosine ribonucleotides de novo biosynthesis
PWY-7234	inosine-5'-phosphate biosynthesis III

PWY-7238	sucrose biosynthesis II
PWY-7560	methylerythritol phosphate pathway II
PWY-7790	UMP biosynthesis II
PWY-7791	UMP biosynthesis III
PWY-7851	coenzyme A biosynthesis II (eukaryotic)
PWY-7953	UDP-N-acetylmuramoyl-pentapeptide biosynthesis III
PWY-922 (eukaryotes and bacteria)	mevalonate pathway I (eukaryotes and bacteria)
PYRIDNUCSAL-PWY	NAD salvage pathway I
SER-GLYSYN-PWY	superpathway of L-serine and glycine biosynthesis I
TRNA-CHARGING-PWY	tRNA charging
UDPNAGSYN-PWY	UDP-N-acetyl-D-glucosamine biosynthesis I
